# Aerobic capacity moderates the association between cervical cord atrophy and clinical disability in mildly disabled multiple sclerosis patients

**DOI:** 10.1177/13524585251318647

**Published:** 2025-02-14

**Authors:** Matteo Albergoni, Paolo Preziosa, Alessandro Meani, Chiara Dallari, Paola Valsasina, Maria A Rocca, Massimo Filippi

**Affiliations:** Neuroimaging Research Unit, Division of Neuroscience, IRCCS San Raffaele Scientific Institute, Milan, Italy; Neuroimaging Research Unit, Division of Neuroscience, IRCCS San Raffaele Scientific Institute, Milan, Italy; Neurology Unit, IRCCS San Raffaele Scientific Institute, Milan, Italy; Vita-Salute San Raffaele University, Milan, Italy; Neuroimaging Research Unit, Division of Neuroscience, IRCCS San Raffaele Scientific Institute, Milan, Italy; Neuroimaging Research Unit, Division of Neuroscience, IRCCS San Raffaele Scientific Institute, Milan, Italy; Neuroimaging Research Unit, Division of Neuroscience, IRCCS San Raffaele Scientific Institute, Milan, Italy; Neuroimaging Research Unit, Division of Neuroscience, IRCCS San Raffaele Scientific Institute, Milan, Italy; Neurology Unit, IRCCS San Raffaele Scientific Institute, Milan, Italy; Vita-Salute San Raffaele University, Milan, Italy; Neuroimaging Research Unit, Division of Neuroscience, IRCCS San Raffaele Scientific Institute, Milan, Italy; Neurology Unit, IRCCS San Raffaele Scientific Institute, Milan, Italy; Vita-Salute San Raffaele University, Milan, Italy; Neurorehabilitation Unit, IRCCS San Raffaele Scientific Institute, Milan, Italy; Neurophysiology Service, IRCCS San Raffaele Scientific Institute, Milan, Italy

**Keywords:** Multiple sclerosis, disability, aerobic capacity, cervical cord, magnetic resonance imaging

## Abstract

**Background:**

Spinal cord volume loss is associated with clinical disability in multiple sclerosis (MS). Aerobic capacity may mitigate the impact of central nervous system (CNS) damage accumulation, exerting beneficial effects on MS-related disability.

**Objectives:**

We investigated whether aerobic capacity could moderate the association between spinal cord atrophy and clinical disability in MS.

**Methods:**

In this cross-sectional analysis, expanded disability status scale (EDSS), peak of oxygen consumption (VO_2_peak), brain volumetric measures, and the normalized mean upper cervical cord area (nMUCCA) were collected from 51 MS patients and 33 healthy controls (HCs). Low aerobic capacity was defined as having a VO_2_peak z-score less than –1.64 standard deviations. In MS patients, we explored whether the association between nMUCCA and EDSS is moderated by the level of aerobic capacity.

**Results:**

The relationship between nMUCCA and EDSS was moderated by aerobic capacity, with a significant nMUCCA × aerobic capacity interaction (β = −0.099, 95% bootstrapped confidence interval [CI] = [−0.172; −0.014], *p* = 0.012). Lower nMUCCA was significantly associated with higher EDSS score in MS patients with low aerobic capacity (β = −0.073, *p* < 0.001), but not in those with high aerobic capacity (β = 0.026, *p* = 0.417).

**Conclusions:**

In MS patients with mild disability, higher aerobic capacity can potentially mitigate the negative impact of spinal cord damage on clinical disability.

## Introduction

Atrophy of the spinal cord occurs from the earliest phases of multiple sclerosis (MS) and is strongly associated with clinical disability and disease progression.^1^ Improvements in magnetic resonance imaging (MRI) technologies have allowed to better evaluate the relevance of spinal cord damage in explaining MS-related disability.^1^ Although spinal cord atrophy is typically evaluated using dedicated MRI sequences,^1^ normalized mean upper cervical cord area (nMUCCA) has been proposed as a reliable measure of spinal cord atrophy, obtainable from high-resolution brain three-dimensional (3D) T1-weighted MRI scans.^2^

Disability can be considered as the result of a complex interplay between an individual’s health condition and contextual factors, such as personal and environmental influences.^3^ Accordingly, several factors could influence the relation between spinal cord damage and disability in MS patients. For instance, psychological personal aspects like depression, or the level of self-efficacy can potentially have an impact on the disability level of MS patients.^4,5^ In addition to psychological aspects, aerobic capacity may represent another important element able to impact the relation between spinal cord neurodegeneration and disability. Aerobic capacity can be viewed as a personal physical resource for MS patients, reflecting their overall health status and potentially mitigating or protecting against the progression of motor impairment associated with MS pathology.^6^ Indeed, aerobic capacity, which represents cardiovascular and respiratory fitness, refers to the ability of the body to utilize oxygen efficiently during prolonged physical activity. The peak of maximum oxygen consumption (VO_2_peak) obtained during a cardiopulmonary exercise testing (CPET) represents the gold standard measure for aerobic capacity. In MS patients, aerobic activity has emerged as a potential lifestyle factor with positive effects on overall fitness, disability, and brain integrity.^7,8^ Some exploratory studies have suggested several hypotheses to explain the mechanisms behind these positive effects, including neuroplasticity,^9^ anti-inflammatory effects,^10,11^ and improved immune regulation,^12,13^ although with conflicting evidence.

Here, we explored whether aerobic capacity may moderate the association between spinal cord atrophy and clinical disability in a well-characterized group of mildly disabled MS patients.

## Methods

### Ethics approval

Approval was received from the institutional ethical standards committee on human experimentation of IRCCS Ospedale San Raffaele for any experiments using human subjects (Protocol number 59/INT/2015). Written informed consent was obtained from all subjects prior to study participation according to the Declaration of Helsinki.

### Study design and population

**Figure 1.**
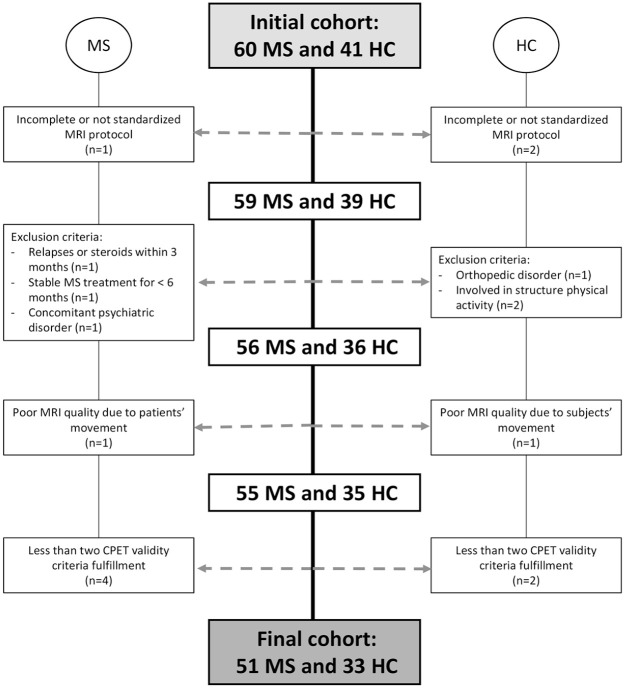
Flow diagram of the study selection process. CPET: cardiopulmonary exercise testing; HC: healthy control; MRI: magnetic resonance imaging; MS: multiple sclerosis.

This study is a secondary retrospective cross-sectional analysis conducted using baseline data obtained from an ongoing interventional rehabilitative protocol at our Institute (ClinicalTrials.gov ID NCT04097418). From the Neuroimaging Research Unit, IRCCS San Raffaele Scientific Institute (Milan, Italy) database, we retrospectively selected 51 consecutive patients with a diagnosis of MS according to the 2017 McDonald criteria and 33 healthy controls (HCs), meeting the following inclusion criteria: 18–65 range of age; no neurological (apart MS), psychiatric, orthopedic, or rheumatological disorders; no concomitant antidepressants, myorelaxants, beta-blockers, psychoactive, or steroids therapies; no drug or alcohol abuse history; no MRI or CPET contraindications.^14^ In addition, patients with MS had to be relapsed and steroid free in the 3 months preceding study enrollment, with a stable disease-modifying treatment for 1 month or more and with an expanded disability status scale (EDSS) score ⩽ 6.0. In accordance with the criteria set by the original study, we excluded all participants engaged in structured physical activities or sport sessions three or more days per week, to eliminate the inclusion of well-trained individuals. The flow diagram of the study selection process is shown in Figure 1.

### Clinical assessment and CPET procedure

Within three days from MRI acquisition, a neurological examination, with EDSS scoring and clinical phenotype definition (relapsing-remitting or progressive MS) was performed by an experienced neurologist blinded to MRI findings. At the same moment, all subjects performed the timed 25-foot walk test (T25FWT) to assess walking speed and the nine-hole peg test (9-HPT) to assess manual dexterity.

CPET was conducted by a cardiologist specialized in cardiac rehabilitation on a recumbent stepper (NuStep T5XR, Ann Arbor, MI, USA) to obtain peak of maximum oxygen consumption (VO_2_peak), considered the gold standard measure of aerobic capacity.^15^ The validity of all CPET exams was carefully evaluated as previously described.^9^ Body mass index (BMI) was calculated for each subject.

Low aerobic capacity was defined as a VO_2_peak z-score at least 1.64 standard deviations below the mean value of HC. Although no specific VO_2_peak thresholds tailored to our study population were identified in the literature, a threshold of –1.64 SD corresponds approximately to the fifth percentile, a commonly used criterion for defining abnormal cases. In this context, this threshold served as a practical and reliable criterion with adequate specificity for classifying patients with low aerobic capacity relative to the physiological levels observed in HCs.

### MRI acquisition

Using a 3.0 Tesla Philips Ingenia CX scanner (Philips Medical Systems), the following brain MRI sequences were acquired with standardized procedures for subjects positioning (receiving Coil = dS-Head-32): (a) axial dual-echo turbo spin echo (TSE), field of view (FOV) = 240 × 240 mm, pixel size = 0.94 × 0.94 mm, 50 slices, 3 mm thick, matrix = 256 × 256, repetition time (TR) = 2599 ms, echo time (TE) = 16/80 ms; (b) 3D T1-weighted magnetization-prepared rapid gradient-echo (MPRAGE), FOV = 256 × 256, pixel size = 1 × 1 mm, 204 slices, 1 mm thick, matrix = 256 × 256, TR = 7 ms, TE = 3.2 ms, TI = 1000 ms, flip angle = 8°.

### MRI analysis

T2-hyperintense white matter (WM) lesion volumes were measured using a local thresholding segmentation technique (Jim 8.0).

After lesion refilling, normalized brain volume (NBV), gray matter volume (NGMV), WM volume (NWMV), and thalamic volume were quantified on 3D T1-weighted images using FSL-SIENAx software and FSL-FIRST tool, applying the scaling factor derived from FSL-SIENAx.

Using the Jim8.0 software, the active surface (AS) method was applied on 3D T1-weighted scans of the brain to derive MUCCA. Briefly, the cord center was manually identified on axial oriented 3D T1-weighted scans by placing landmarks at the extremes of the studied cord region (i.e. the cranial limit of the odontoid process and the caudal border of C2) and approximately every 10 mm between these landmarks. These markers were used by an automated procedure to define cord contours. The mean MUCCA was obtained as the total cord volume divided by the cord length. Normalized MUCCA (nMUCCA) was obtained adjusting for FSL SIENAx brain scaling factor, inversely proportional to the total intracranial volume, according to the formula previously reported.^16^

### Statistical analysis

Demographic, clinical, and MRI data differences between patients and HCs, between MS patients with low or high aerobic capacity, and according to disease phenotype were tested using Chi-square, two-sample *t*, Mann–Whitney test, and one-way analysis of variance (ANOVA) test with Bonferroni post hoc group comparisons, as appropriate. T2-hyperintense WM LV were log-transformed. The normality of clinical and MRI variables was assessed using the Shapiro–Wilk test. In MS patients, we performed Spearman’s rank correlations between clinical measures (EDSS score, T25FWT and 9-HPT) and brain volumetric MRI measures and nMUCCA. Correlation analyses were also performed separately for MS patients with low and high aerobic capacity. Finally, we conducted a 5000 bootstrap samples moderation analysis corrected for age and sex considering nMUCCA as independent variable, EDSS as dependent variable and aerobic capacity as moderator. Analyses were performed using SPSS software (version 28.0, PROCESS Macro SPSS). The *p*-values < 0.05 were deemed statistically significant.

## Results

### Demographic and MRI findings

Compared to HCs, MS patients were significantly older (47 vs 44 years of age, *p* < 0.001) whereas sex and BMI were not significantly different (*p* ⩾ 0.560; see Table 1). MS patients had significantly worse T25FWT and 9-HPT performance (*p* < 0.001), and they had significantly higher brain T2-hyperintense WM LV (*p* < 0.001), as well as significantly lower NBV, NGMV, NWMV, thalamic volume, and nMUCCA (all *p* < 0.001; see Table 1).

**Table 1.** Comparison of demographic, clinical, and MRI characteristics between HCs and MS patients, as well as between MS with low and high aerobic capacity.

	HC (33)	All MS patients (51)	*p*-value	MS patients with low aerobic capacity (31)	MS patients with high aerobic capacity (20)	*p*-value
Age mean (SD; (years)	44.52 (11.31)	47.12 (7.65)	< 0.001^a^	48.17 (6.86)	45.50 (8.82)	0.229^a^
Sex (male/female; (*n*)	9/24	17/34	0.560^b^	9/22	8/12	0.417^b^
BMI mean (SD)	22.94 (3.52)	23.08 (3.97)	0.864^a^	24.42 (3.95)	21.07 (3.26)	0.003^a^
EDSS median (IQR)	—	2.5 (2.0;4.0)	—	3.0 (2.0;4.0)	2.5 (2.0;3.0)	0.355^c^
T25FWT median (IQR; (seconds)	4.14 (3.77;4.43)	5.07 (4.61;5.82)	< 0.001^c^	5.39 (4.60;5.30)	4.94 (4.62;5.30)	0.116^c^
9-HPT median (IQR; seconds)	18.82 (17.00;20.70)	24.32 (21.34;28.68)	< 0.001^c^	24.60 (21.34;29.41)	24.29 (21.16;27.65)	0.780^c^
Disease duration mean (SD; years)	—	14.1 (8.2)	—	15.43 (7.56)	11.63 (8.62)	0.065^a^
Phenotypes RR/P (*n*)	—	38/13	—	23/8	15/5	0.949^b^
DMT: None/first line/second line (n)	—	6/23/22	—	3/13/15	3/10/7	0.615^b^
VO_2_peak mean (SD; mL/kg/min)	28.11 (6.04)	18.01 (4.38)	< 0.001^a^	15.36 (2.51)	22.13 (3.34)	< 0.001^a^
zVO_2_peak mean (SD)	0.00 (1.00)	−1.67 (0.72)	< 0.001^a^	−2.11 (0.42)	−0.99 (0.55)	< 0.001^a^
T2-hyperintense WM LV median (IQR; mL)	0.00 (0.00;0.01)	3.67 (3.34;3.96)	< 0.001^c^	3.71 (3.41;3.96)	3.60 (2.93;3.95)	0.643^c^
NBV mean (SD; mL)	1571 (38)	1492 (57)	< 0.001^a^	1484 (60)	1501 (51)	0.340^a^
NGMV mean (SD; mL)	885 (35)	836 (33)	< 0.001^a^	833 (33)	843 (34)	0.291^a^
NWMV mean (SD; mL)	686 (22)	655 (39)	< 0.001^a^	654 (44)	659 (32)	0.611^a^
Thalamic volume mean (SD; mL)	22.26 (1.35)	19.72 (2.23)	< 0.001^a^	19.44 (2.32)	20.14 (2.05)	0.267^a^
nMUCCA mean (SD; mm^2^)	82.13 (6.70)	75.86 (8.01)	< 0.001^a^	75.73 (8.62)	76.05 (7.17)	0.888^a^

9-HPT: nine hole peg test; BMI: body mass index; DMT: disease-modifying therapy; EDSS: expanded disability status scale; HC: healthy control; IQR: interquartile range; kg: kilograms; min: minutes; mL: milliliters; mm^2^: square millimeters; MRI: magnetic resonance imaging; MS: multiples sclerosis; *n*: number; NBV: normalized brain volume; NGMV: normalized gray matter volume; nMUCCA: normalized mean upper cervical cord area; NWMV: normalized white matter volume; P: progressive phenotype; RR: relapsing-remitting phenotype; SD: standard deviation; T25FWT: timed 25-foot walk test; VO_2_peak: peak of maximal oxygen consumption; WM LV: white matter lesion volume.

a *t*-test.

b Pearson’s Chi-square test.

c Mann–Whitney test.

Compared to relapsing-remitting MS patients, those with a progressive disease were more frequently males, had significantly higher EDSS score (*p* = 0.012), worse T25FWT and 9-HPT performance (*p* ⩽ 0.024). VO_2_peak values or MRI data were not significantly different between the two groups (see Supplementary Table 1).

### CPET findings

CPET reliability criteria have been proposed within the context of MS population to ensure consistent and reproducible results during exercise testing especially in patients with mild disability, considering the physiological and functional challenges associated with this disease.^17^

In all HC subjects considered for this secondary analysis, a minimum of three out of four CPET criteria were consistently fulfilled. Specifically, the VO_2_ plateau criterion was achieved in 75% of the sample, the respiratory exchange ratio (RER) ⩾ 1.10 criterion in 100%, the rating of perceived exertion (RPE) measured on the Borg Scale ⩾ 17 criterion in 100%, and the HR criterion in 92%.

In all MS patients considered for this secondary analysis, a minimum of two out of four CPET criteria were consistently fulfilled. Specifically, the VO_2_ plateau criterion was achieved in 53% of the sample, the RER ⩾ 1.10 criterion in 63%, the RPE ⩾ 17 criterion in 87%, and the HR criterion in 17%. Compared to HC, MS patients had a lower value of zVO_2_peak (*p* < 0.001). Thirty-one (61%) patients were characterized by low aerobic capacity. Compared to MS patients with high aerobic capacity, those with low aerobic capacity showed significantly higher BMI values (*p* ⩽ 0.003), no other demographic, clinical and MRI measures differences were found (*p* ⩾ 0.065; see Table 1).

### Associations between clinical and MRI measures

**Figure 2.**
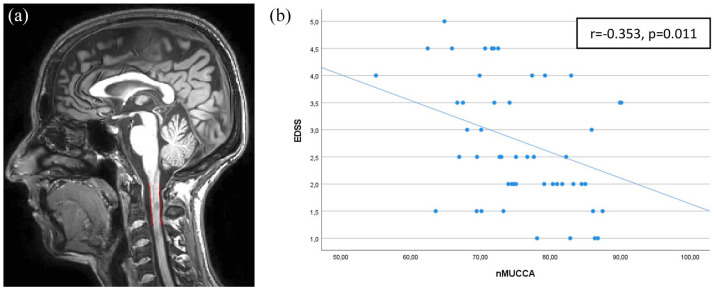
Segmentation of MUCCA measure on (a) 3D T1-weighted image and (b) scatter plot within the MS group highlighting the association between EDSS and nMUCCA. EDSS: expanded disability status scale; nMUCCA: normalized mean upper cervical cord area; *p: p*-value; *r: r*-value.

In MS patients, a higher EDSS score was significantly associated with lower nMUCCA (*r* = −0.353, *p* = 0.011; see Figure 2, Table 2). Such an association remained statistically significant only for MS patients with low aerobic capacity (*r* = −0.536, *p* = 0.002; see Supplementary Table 2). Worse 9-HPT performance was significantly associated with lower NBV, NGMV, and thalamic volume (*r* values from −0.294 to −0.466, *p* ⩽ 0.027), whereas the association with nMUCCA was not statistically significant (*p* = 0.154; see Table 2). T25FWT performance showed no significant association with brain and cord MRI findings (see Table 2).

**Table 2.** Spearman rank correlations between clinical and MRI measures in MS patients.

	T2-hyperintense WM LV	NBV	NGMV	NWMV	Thalamic volume	nMUCCA
EDSS *r* (*p*-value)	0.132 (0.358)	−0.033 (0.852)	−0.059 (0.683)	−0.008 (0.957)	−0.228 (0.107)	−**0.353 (0.011)**
T25FWT *r* (*p*-value)	0.246 (0.082)	0.068 (0.633)	−0.001 (0.993)	0.062 (0.666)	−0.187 (0.190)	−0.215 (0.129)
9-HPT *r* (*p*-value)	0.273 (0.052)	−**0.294 (0.027)**	−**0.309 (0.027)**	−0.223 (0.116)	−**0.466 (< 0.001)**	−0.203 (0.154)

9-HPT: nine-hole peg test; EDSS: expanded disability status scale; NBV: normalized brain volume; NGMV: normalized gray matter volume; nMUCCA: normalized mean upper cervical cord area; NWMV: normalized white matter volume; *r: r*-value; T25FWT: timed 25-foot walk test; WM LV: white matter lesion volume.

Statistically significant associations are shown in bold.

### Moderation analysis

In MS patients, VO_2_peak and nMUCCA were normally distributed, as confirmed by the Shapiro–Wilk test (*p* = 0.206 and *p* = 0.486, respectively), while the EDSS was not normally distributed (*p* = 0.006). Therefore, we decided to conduct the moderation analysis using bias corrected and accelerated 5000-sample bootstrapping.

**Figure 3.**
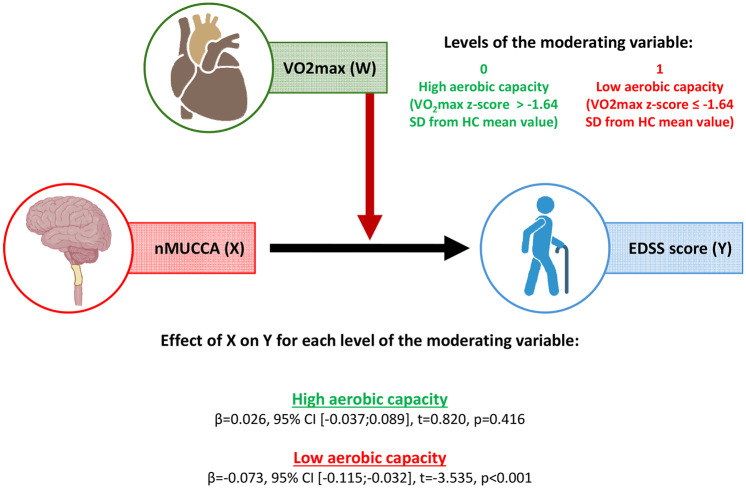
Moderation analysis corrected for age and sex performed in MS patients considering EDSS as outcome variable, nMUCCA as predictor and aerobic capacity as moderator. β: beta value; EDSS: expanded disability status scale; MS: multiple sclerosis; nMUCCA: normalized mean upper cervical cord area; *p: p*-value; SD: standard deviation; *t: t*-value; VO_2_peak: peak of maximal oxygen consumption; W: moderator variable; X: the independent variable; Y: the dependent variable; 95% CI: 95% confidence interval.

In MS patients, the interaction between nMUCCA and aerobic capacity was significant (β = −0.099, 95% bootstrapped confidence interval [CI] = [−0.172; −0.014], *p* = 0.012), indicating that the relationship between nMUCCA and EDSS was moderated by aerobic capacity. Lower nMUCCA was significantly associated with higher EDSS score only in MS patients with low aerobic capacity (β = −0.073, 95% CI = [−0.115; −0.032], *p* < 0.001), whereas no significant association was found in MS patients with high aerobic capacity (β = 0.026, 95% CI = [−0.037; 0.089], *p* = 0.417; see Figure 3).

## Discussion

We found that, compared to HC, MS patients showed a significant lower nMUCCA,^18–20^ which was significantly associated with more severe disability,^18–20^ thus supporting the relevance of spinal cord volume loss in determining clinical impairment.^1^ Moreover, in line with previous studies,^21^ we found that MS patients had significantly lower aerobic capacity compared to HC, suggesting a state of physical deconditioning.

Our results suggest that aerobic capacity may influence the association between spinal cord atrophy and clinical disability. We found a significant interaction between nMUCCA and aerobic capacity, along with the evidence that nMUCCA was negatively associated with EDSS scores only in MS patients with low aerobic capacity. Considering that no significant differences were found in demographic, clinical, and MRI measures between MS patients with low or high aerobic capacity, except for a significantly lower BMI in those with higher aerobic capacity, these findings suggest that higher aerobic capacity may mitigate the detrimental impact of irreversible spinal cord volume loss on MS-related clinical disability. In contrast, in MS patients with lower aerobic capacity, the severity of spinal cord damage has a more pronounced impact on clinical disability. Therefore speculating, a higher aerobic capacity may represent a personal physical resource useful for reducing the impact of spinal cord damage. Indeed, better cardiovascular fitness seems to be associated with better brain health through multiple mechanisms. Although no univocal evidence has been published within the MS field, aerobic exercise (which usually results in better aerobic capacity) may lead to improvements in the cerebral blood flow,^22,23^ to the promotion of neuroplasticity^9,24^ and stimulation of brain-derived neurotrophic factors (BDNFs).^25^ In addition, better aerobic capacity may positively influence mood and fatigue, as well as reduce stress, anxiety, and depressive symptoms.^12,26–28^

Moreover, aerobic exercise may have potential direct effect not only on the brain but also on the spinal cord itself.^29^ Some experimental studies seem to suggest that exercise may reduce immune cell infiltration, including T cells, macrophages, and B cells, and decreases markers of inflammation, oxidative stress, and axonal damage. It also preserves motor neurons in the lumbar spinal cord and enhances myelin integrity. These changes are linked to exercise-induced modulation of inflammatory cytokines, such as interferon gamma (IFN-γ) and interleukin-17 (IL-17), and reduced blood–brain barrier permeability.^29^ Moreover, exercise influences neurotrophic factors, such as BDNF and nerve growth factor (NGF), which support neural plasticity and repair in the spinal cord and brain.^29^ Further investigations are needed to better understand the underling mechanisms and potential therapeutic targets of aerobic exercise and active lifestyle.

Unexpectedly,^19^ we found no significant association between brain volumetric MRI measures and EDSS score, or T25FWT performance. Similar results were obtained when the analysis was stratified by MS patients with low and high aerobic capacity. These findings may be explained by the evaluation of MS patients with relatively mild disability, limited variability in EDSS scores, and a narrow range of walking speed, combined with a global rather than regional assessment of brain tissue loss. On the contrary, 9-HPT performance was associated with brain volumetric measures, potentially representing a more appropriate clinical measure of neurodegeneration in these early phases of the disease. However, we confirmed the more relevant role of spinal cord rather than brain damage in explaining disability.^1,30^ Nonetheless, since our study included MS patients with mild disability, our results should be interpreted with caution. Accordingly, they cannot be generalized to all MS patients and require confirmation in future studies involving larger cohorts, particularly those with higher levels of disability.

Our study has several limitations. Although age has been shown to influence VO_2_peak values, with a gradual decline across the lifespan similarly observed in both HC and MS patients,^31^ we did not apply any age-related correction when calculating zVO_2_peak for determining aerobic capacity levels. Unfortunately, previously published data utilized a different protocol compared to our study for determining VO_2_peak values, and our small cohort of HC did not allow for a reliable estimation of the age effect on VO_2_peak. As a result, some older MS patients may have been wrongly classified as having low aerobic capacity, despite being comparable to age-matched HC. This could explain the relatively high prevalence (61%) of MS patients classified as having low aerobic capacity, despite their relatively low disability level (median EDSS score = 2.5). Due to the design of the study, HC and MS patients who regularly engage in sports or physical activity were not included. Moreover, detailed information regarding the amount of sport participation, exercise habits, and lifestyle was not collected. Therefore, this study mainly explored the impact of an active lifestyle, rather than the effects of physical activity. Future studies that incorporate a comprehensive assessment of physical activity and lifestyle, as well as MS patients who regularly engage in physical activity, could provide further insights into the beneficial effects of cardiovascular fitness in mitigating the detrimental impact of spinal cord damage on clinical disability.

Finally, the cross-sectional nature of our study precluded direct assessment of whether longitudinal changes in aerobic capacity (perhaps obtained directly through an aerobic training period) could impact the relationship between nMUCCA and EDSS score, as would be possible with an interventional longitudinal design. Moreover, due to the cross-sectional design of this study, causality between a high level of aerobic capacity and low disability, as suggested by our data, cannot be established. MS patients with lower levels of disability are more likely to engage in physical activity, which could, in turn, contribute to higher aerobic capacity. However, in the direct comparison, we found that while progressive MS patients had significantly higher EDSS scores compared to relapsing-remitting MS patients, no significant between-group difference in VO_2_peak values was found. These additional findings suggest that aerobic capacity may be, at least partially independent from the severity of clinical disability in MS patients. Nevertheless, further studies, particularly longitudinal ones, are required to better explore this association.

In conclusion, our study showed that a higher aerobic capacity may act as a personal physical resource able to reduce the impact of spinal cord damage on clinical disability in MS patients. For this reason, our study supports the importance of aerobic exercise and regular physical activity aimed at improving aerobic capacity in MS patients.^32,33^ This approach can be a rewarding strategy complementing specific pharmacologic therapies to improve MS patients’ management and their global health.^32,33^

## Supplemental Material

sj-docx-1-msj-10.1177_13524585251318647 – Supplemental material for Aerobic capacity moderates the association between cervical cord atrophy and clinical disability in mildly disabled multiple sclerosis patients Supplemental material, sj-docx-1-msj-10.1177_13524585251318647 for Aerobic capacity moderates the association between cervical cord atrophy and clinical disability in mildly disabled multiple sclerosis patients by Matteo Albergoni, Paolo Preziosa, Alessandro Meani, Chiara Dallari, Paola Valsasina, Maria A Rocca and Massimo Filippi in Multiple Sclerosis Journal

sj-docx-2-msj-10.1177_13524585251318647 – Supplemental material for Aerobic capacity moderates the association between cervical cord atrophy and clinical disability in mildly disabled multiple sclerosis patients Supplemental material, sj-docx-2-msj-10.1177_13524585251318647 for Aerobic capacity moderates the association between cervical cord atrophy and clinical disability in mildly disabled multiple sclerosis patients by Matteo Albergoni, Paolo Preziosa, Alessandro Meani, Chiara Dallari, Paola Valsasina, Maria A Rocca and Massimo Filippi in Multiple Sclerosis Journal

sj-docx-3-msj-10.1177_13524585251318647 – Supplemental material for Aerobic capacity moderates the association between cervical cord atrophy and clinical disability in mildly disabled multiple sclerosis patients Supplemental material, sj-docx-3-msj-10.1177_13524585251318647 for Aerobic capacity moderates the association between cervical cord atrophy and clinical disability in mildly disabled multiple sclerosis patients by Matteo Albergoni, Paolo Preziosa, Alessandro Meani, Chiara Dallari, Paola Valsasina, Maria A Rocca and Massimo Filippi in Multiple Sclerosis Journal

## References

[1] Rocca MA Preziosa P Filippi M . What role should spinal cord MRI take in the future of multiple sclerosis surveillance? Expert Rev Neurother 2020; 20(8): 783–797. 32133874 10.1080/14737175.2020.1739524

[2] Liu Y Lukas C Steenwijk MD , et al. Multicenter validation of mean upper cervical cord area measurements from head 3D T1-weighted MR imaging in patients with multiple sclerosis. AJNR Am J Neuroradiol 2016; 37(4): 749–754. 26659338 10.3174/ajnr.A4635 PMC7960152

[3] Ramari C Awadia Z Bansi J , et al. The MoxFo initiative—outcomes: Outcome measures in studies of exercise training in multiple sclerosis; scoping review of reviews and classification according to the ICF framework. Mult Scler 2023; 29(13): 1578–1594. 37880966 10.1177/13524585231204451

[4] Dymecka J Gerymski R Tataruch R , et al. Fatigue, physical disability and self-efficacy as predictors of the acceptance of illness and health-related quality of life in patients with multiple sclerosis. Int J Environ Res Public Health 2021; 18: 13237. 34948845 10.3390/ijerph182413237 PMC8703876

[5] Young CA Langdon D Rog D , et al. Prevalence, treatment and correlates of depression in multiple sclerosis. Mult Scler Relat Disord 2024; 87: 105648. 38713965 10.1016/j.msard.2024.105648

[6] O’Brien C Holtzer R . Physical reserve: Construct development and predictive utility. Aging Clin Exp Res 2023; 35(5): 1055–1062. 36848030 10.1007/s40520-023-02371-5

[7] Prakash RS Snook EM Motl RW , et al. Aerobic fitness is associated with gray matter volume and white matter integrity in multiple sclerosis. Brain Res 2010; 1341: 41–51. 19560443 10.1016/j.brainres.2009.06.063 PMC2884046

[8] Proschinger S Kuhwand P Rademacher A , et al. Fitness, physical activity, and exercise in multiple sclerosis: A systematic review on current evidence for interactions with disease activity and progression. J Neurol 2022; 269(6): 2922–2940. 35084560 10.1007/s00415-021-10935-6 PMC9119898

[9] Morozumi T Preziosa P Meani A , et al. Influence of cardiorespiratory fitness and MRI measures of neuroinflammation on hippocampal volume in multiple sclerosis. J Neurol Neurosurg Psychiatry 2023; 95: 29–36. 37468307 10.1136/jnnp-2023-331482

[10] Wong VL Holahan MR . A systematic review of aerobic and resistance exercise and inflammatory markers in people with multiple sclerosis. Behav Pharmacol 2019; 30(8): 653–660. 31703029 10.1097/FBP.0000000000000514

[11] Negaresh R Motl RW Mokhtarzade M , et al. Effects of exercise training on cytokines and adipokines in multiple sclerosis: A systematic review. Mult Scler Relat Disord 2018; 24: 91–100. 29982111 10.1016/j.msard.2018.06.008

[12] Motl RW Pilutti LA . The benefits of exercise training in multiple sclerosis. Nat Rev Neurol 2012; 8: 487–497. 22825702 10.1038/nrneurol.2012.136

[13] Einstein O Katz A Ben-Hur T . Physical exercise therapy for autoimmune neuroinflammation: Application of knowledge from animal models to patient care. Autoimmun Rev 2022; 21(4): 103033. 34995760 10.1016/j.autrev.2022.103033

[14] American Thoracic Society and American College of Chest Physicians. ATS/ACCP statement on cardiopulmonary exercise testing. Am J Respir Crit Care Med 2003; 167: 211–277. 12524257 10.1164/rccm.167.2.211

[15] Stickland MK Butcher SJ Marciniuk DD , et al. Assessing exercise limitation using cardiopulmonary exercise testing. Pulm Med 2012; 2012: 824091. 23213518 10.1155/2012/824091 PMC3506917

[16] Horsfield MA Sala S Neema M , et al. Rapid semi-automatic segmentation of the spinal cord from magnetic resonance images: Application in multiple sclerosis. NeuroImage 2010; 50: 446–455. 20060481 10.1016/j.neuroimage.2009.12.121 PMC2830007

[17] Langeskov-Christensen M Langeskov-Christensen D Overgaard K , et al. Validity and reliability of VO(2)-max measurements in persons with multiple sclerosis. J Neurol Sci 2014; 342: 79–87. 24825731 10.1016/j.jns.2014.04.028

[18] Daams M Weiler F Steenwijk MD , et al. Mean upper cervical cord area (MUCCA) measurement in long-standing multiple sclerosis: Relation to brain findings and clinical disability. Mult Scler 2014; 20(14): 1860–1865. 24812042 10.1177/1352458514533399

[19] de Ruiter LRJ Loonstra FC Jelgerhuis JR , et al. Association of volumetric MRI measures and disability in MS patients of the same age: Descriptions from a birth year cohort. Mult Scler Relat Disord 2023; 71: 104568. 36805177 10.1016/j.msard.2023.104568

[20] Weeda MM Zywicki S Brouwer I , et al. Upper cervical cord atrophy is independent of cervical cord lesion volume in early multiple sclerosis: A two-year longitudinal study. Mult Scler Relat Disord 2022; 60: 103713. 35272146 10.1016/j.msard.2022.103713

[21] Langeskov-Christensen M Heine M Kwakkel G , et al. Aerobic capacity in persons with multiple sclerosis: A systematic review and meta-analysis. Sports Med 2015; 45(6): 905–923. 25739555 10.1007/s40279-015-0307-x

[22] Lefferts WK Rosenberg AJ Schroeder EC , et al. Assessment of cerebrovascular dynamics and cognitive function with acute aerobic exercise in persons with multiple sclerosis. Int J MS Care 2021; 23(4): 162–169. 34483755 10.7224/1537-2073.2020-003 PMC8405144

[23] Kleinloog JPD Mensink RP Ivanov D , et al. Aerobic exercise training improves cerebral blood flow and executive function: A randomized, controlled cross-over trial in sedentary older men. Front Aging Neurosci 2019; 11: 333. 31866855 10.3389/fnagi.2019.00333 PMC6904365

[24] Stellmann JP Maarouf A Schulz KH , et al. Aerobic exercise induces functional and structural reorganization of CNS networks in multiple sclerosis: A randomized controlled trial. Front Hum Neurosci 2020; 14: 255. 32714172 10.3389/fnhum.2020.00255 PMC7340166

[25] Mackay CP Kuys SS Brauer SG . The effect of aerobic exercise on brain-derived neurotrophic factor in people with neurological disorders: A systematic review and meta-analysis. Neural Plast 2017; 2017: 4716197. 29057125 10.1155/2017/4716197 PMC5625797

[26] Carter A Daley A Humphreys L , et al. Pragmatic intervention for increasing self-directed exercise behaviour and improving important health outcomes in people with multiple sclerosis: A randomised controlled trial. Mult Scler 2014; 20(8): 1112–1122. 24421303 10.1177/1352458513519354

[27] Feys P Moumdjian L Van Halewyck F , et al. Effects of an individual 12-week community-located “start-to-run” program on physical capacity, walking, fatigue, cognitive function, brain volumes, and structures in persons with multiple sclerosis. Mult Scler 2019; 25(1): 92–103. 29113572 10.1177/1352458517740211

[28] Albergoni M Pagani E Preziosa P , et al. Thalamic nuclei volume partially mediates the effects of aerobic capacity on fatigue in people with multiple sclerosis. J Neurol 2024; 271(6): 3378–3388. 38507073 10.1007/s00415-024-12277-5

[29] Prosperini L Di Filippo M . Beyond clinical changes: Rehabilitation-induced neuroplasticity in MS. Mult Scler 2019; 25(10): 1348–1362. 31469359 10.1177/1352458519846096

[30] Morozumi T Preziosa P Meani A , et al. Brain and cervical spinal cord MRI correlates of sensorimotor impairment in patients with multiple sclerosis. Mult Scler 2024; 30(8): 1004–1015. 38912804 10.1177/13524585241260145

[31] Schlagheck ML Bansi J Langeskov-Christensen M , et al. Cardiorespiratory fitness (V۟O(2peak)) across the adult lifespan in persons with multiple sclerosis and matched healthy controls. J Sci Med Sport 2024; 27(1): 10–15. 10.1016/j.jsams.2023.10.009 37951825

[32] Halabchi F Alizadeh Z Sahraian MA , et al. Exercise prescription for patients with multiple sclerosis; potential benefits and practical recommendations. BMC Neurol 2017; 17: 185. 28915856 10.1186/s12883-017-0960-9 PMC5602953

[33] Motl RW . Exercise and Multiple Sclerosis. Adv Exp Med Biol 2020; 1228: 333–343. 32342468 10.1007/978-981-15-1792-1_22

