# Supplementary material for: Aerobic capacity moderates the association between cervical cord atrophy and clinical disability in mildly disabled multiple sclerosis patients
Source: Mult Scler. 2025 Feb 14;31(5):558–67. doi: 10.1177/13524585251318647 (PMC12008468; doi:10.1177/13524585251318647)
Supplement: sj-docx-1-msj-10.1177_13524585251318647 – Supplemental material for Aerobic capacity moderates the association between cervical cord atrophy and clinical disability in mildly disabled multiple sclerosis patients [file sj-docx-1-msj-10.1177_13524585251318647.docx]

MS Journal Appendix for MRI methodology

| **Hardware** | |
| --- | --- |
| Field strength | 3.0 T |
| Manufacturer | Philips Medical Systems |
| Model | Philips Ingenia CX |
| Coil type  (e.g. head, surface) | dS-Head-32 |
| Number of coil channels | 32 |

| **Acquisition sequence** | | |
| --- | --- | --- |
| Type  (e.g. FLAIR, DIR, DTI, fMRI) | dual-echo turbo spin echo (TSE) | |
| Acquisition time | 7.21 min | |
| Orientation | axial | |
| Alignment  (e.g. anterior commissure/poster commissure line | AC-PC line | |
| Voxel size | 0.94x0.94x3 mm | |
| TR | 2599 ms | |
| TE | 16,80 ms | |
| TI | - | |
| Flip angle | 90 | |
| NEX | 1 | |
| Field of view | 240x240 mm | |
| Matrix size | 256x256 | |
| Parallel imaging | Yes | No |
| If used, parallel imaging method:  (e.g. SENSE, GRAPPA) | - | |
| Cardiac gating | Yes | No |
| If used, cardiac gating method:  (e.g. PPU or ECG) |  | |
| Contrast enhancement | Yes | No |
| **Acquisition sequence** | | |
| If used, provide name of contrast agent, dose and timing of scan post-contrast administration |  | |
| Other parameters:  Echo train length | 6 | |

| **Acquisition sequence** | | |
| --- | --- | --- |
| Type  (e.g. FLAIR, DIR, DTI, fMRI) | MPRAGE | |
| Acquisition time | 8.53 min | |
| Orientation | Sagittal | |
| Alignment  (e.g. anterior commissure/poster commissure line) | None | |
| Voxel size | 1x1x1 mm | |
| TR | 7 ms | |
| TE | 3.2 ms | |
| TI | 1000 ms | |
| Flip angle | 8° | |
| NEX | 1 | |
| Field of view | 256x256 mm | |
| Matrix size | 256x256 | |
| Parallel imaging | Yes | No |
| If used, parallel imaging method:  (e.g. SENSE, GRAPPA) | N.A. | |
| Cardiac gating | Yes | No |
| If used, cardiac gating method:  (e.g. PPU or ECG) |  | |
| Contrast enhancement | Yes | No |

| **Image analysis methods and outputs** | |
| --- | --- |
| ***Brain lesions*** | |
| Type  (e.g. Gd-enhancing, T2-hyperintense, T1-hypointense) | T2-hyperintense |
| Analysis method | local thresholding segmentation technique |
| Analysis software | XINAPSE-Jim 8.0 |
| Output measure  (e.g. count or volume [ml]) | Volume [ml] |
| ***Brain tissue volumes*** | |
| Type  (e.g. whole brain, grey matter, white matter, spinal cord) | Whole brain, cortical, deep gray matter, white matter |
| Analysis method | Fully automated tissue segmentation |
| Analysis software | FSL-SIENAx, FIRST, XINAPSE-Jim 8.0 |
| Output measure  (e.g. absolute tissue volume in ml, tissue volume as a fraction of intracranial volume, percentage change in tissue volumes) | Tissue volume in ml normalized for head size |
| ***Cervical Cord Atrophy*** | |
| Type  (e.g. whole brain, grey matter, white matter, spinal cord) | Mean Upper Cervical Cord Area |
| Analysis method | Active surface method for total cross-sectional area |
| Analysis software | Jim 8; Xinapse Systems, Colchester, England |
| Output measure  (e.g. absolute tissue volume in ml, tissue volume as a fraction of intracranial volume, percentage change in tissue volumes) | Cross sectional area in mm^2^ normalized for brain scaling factor |
| ***Tissue measures (e.g. MTR, DTI, T1-RT, T2-RT, T2*, T2’, ^1^H-MRS, perfusion, Na)*** | |
| Type  (e.g. whole brain, grey matter, white matter, spinal cord, normal-appearing grey matter or white matter) | N.A. |
| Analysis method | N.A. |
| Analysis software | N.A. |
| Output measure | N.A. |
| ***Other MRI measures (e.g. functional MRI)*** | |
| Type  (e.g. whole brain, grey matter, white matter, spinal cord, normal-appearing grey matter or white matter) | N.A. |
| Analysis method | N.A. |
| Analysis software | N.A. |
| Output measure | N.A. |
