# Supplementary material for: Aerobic capacity moderates the association between cervical cord atrophy and clinical disability in mildly disabled multiple sclerosis patients
Source: Mult Scler. 2025 Feb 14;31(5):558–67. doi: 10.1177/13524585251318647 (PMC12008468; doi:10.1177/13524585251318647)
Supplement: sj-docx-2-msj-10.1177_13524585251318647 – Supplemental material for Aerobic capacity moderates the association between cervical cord atrophy and clinical disability in mildly disabled multiple sclerosis patients [file sj-docx-2-msj-10.1177_13524585251318647.docx]

**Supplementary Table 1.** Comparison of demographic and clinical data between HC and MS phenotypes.

|  | **HC**  **(33)** | **RRMS**  **(38)** | **PMS**  **(13)** | **HC vs RRMS**  **p-value** | **HC vs PMS**  **p-value** | **RRMS vs PMS**  **p-value** |
| --- | --- | --- | --- | --- | --- | --- |
| **Age** Mean (SD) [years]^a^ | 44.52 (11.31) | 46.97 (7.52) | 47.54 (8.34) | 0.003 ^a^ | 0.030 ^a^ | 1.000 ^a^ |
| **Sex** (Male/Female) [n]^b^ | 9/24 | 9/29 | 8/5 | 0.729 ^b^ | 0.030 ^b^ | 0.012 ^b^ |
| **BMI** Mean (SD)^a^ | 22.94 (3.52) | 22.72 (4.03) | 24.17 (3.71) | 1.000 ^a^ | 0.981 ^a^ | 0.714 ^a^ |
| **EDSS** Median (IQR)^c^ | - | 2.5 (2.0;3.0) | 4.0 (3.5;4.5) | - | - | <0.001 ^c^ |
| **T25FWT** Median (IQR) [seconds] | 4.14  (3.77;4.43) | 4.89  (4.51;5.39) | 6.07  (5.30;6.41) | <0.001 ^a*^ | <0.001 ^a*^ | 0.007 ^a*^ |
| **9-HPT** Median (IQR) [seconds] | 18.82 (17.00;20.70) | 23.51 (19.31;27.51) | 26.81 (24.65;30.77) | <0.001 ^a*^ | <0.001 ^a*^ | 0.024 ^a*^ |
| **Disease duration** Mean (SD) [years]^d^ | - | 14.96 (8.49) | 11.70 (7.03) | - | - | 0.220 ^d^ |
| **DMT:** None/first line/second line [n] | - | 4/19/15 | 2/4/7 | - | - | 0.484 ^b^ |
| **VO2peak** Mean (SD) [mL/Kg/min]^a^ | 28.11 (6.04) | 17.96 (4.04) | 18.16 (5.43) | <0.001 ^a^ | <0.001 ^a^ | 1.000 ^a^ |
| **zVO2peak** Mean (SD) | 0.00 (1.00) | -1.68 (0.67) | -1.65 (0.90) | <0.001 ^a^ | <0.001 ^a^ | 1.000 ^a^ |
| **T2-hyperintense WM LV** Median (IQR) [mL] | 0.00 (0.00;0.01) | 3.69 (3.13;3.97) | 3.57 (3.54;3.79) | <0.001 ^c^ | <0.001 ^c^ | 0.931 ^c^ |
| **NBV** Mean (SD) [mL] | 1571 (38) | 1492 (57) | 1492 (61) | <0.001 ^a^ | <0.001 ^a^ | 1.000 ^a^ |
| **NGMV** Mean (SD) [mL] | 885 (35) | 837 (34) | 834 (34) | <0.001 ^a^ | <0.001 ^a^ | 1.000 ^a^ |
| **NWMV** Mean (SD) [mL] | 686 (22) | 655 (40) | 658 (39) | <0.001 ^a^ | 0.042 ^a^ | 1.000 ^a^ |
| **Thalamic volume** Mean (SD) [mL] | 22.26 (1.35) | 19.78 (2.40) | 19.53 (1.69) | <0.001 ^a^ | <0.001 ^a^ | 1.000 ^a^ |
| **nMUCCA** Mean (SD) [mm^2^] | 82.13 (6.70) | 76.72 (7.58) | 73.34 (9.02) | 0.009 ^a^ | 0.002 ^a^ | 0.492 ^a^ |

^a^ANOVA-with Bonferroni post-hoc test for group comparisons; ^b^Pearson’s Chi-square test; ^c^Mann-Whitney test; ^d^t-test; *analysis performed on the inverse measure to obtain a normal distribution.

Abbreviations: 9-HPT=nine-hole peg test; BMI=body mass index; EDSS=expanded disability status scale; HC=healthy controls; IQR=interquartile range; Kg= kilograms; min=minutes; mL=milliliters; mm^2^=square millimeters; MS=multiples sclerosis; n=number PMS=progressive phenotype; RRMS=relapsing remitting phenotype; SD=standard deviation; T25FWT=timed 25-foot walk test; VO2peak=peak of maximal oxygen consumption.
