# Supplementary material for: Aerobic capacity moderates the association between cervical cord atrophy and clinical disability in mildly disabled multiple sclerosis patients
Source: Mult Scler. 2025 Feb 14;31(5):558–67. doi: 10.1177/13524585251318647 (PMC12008468; doi:10.1177/13524585251318647)
Supplement: sj-docx-3-msj-10.1177_13524585251318647 – Supplemental material for Aerobic capacity moderates the association between cervical cord atrophy and clinical disability in mildly disabled multiple sclerosis patients [file sj-docx-3-msj-10.1177_13524585251318647.docx]

**Supplementary Table 2.** Spearman Rank Correlations between clinical and MRI measures in MS with low or high aerobic capacity. Statistically significant associations are shown in bold.

|  |  | **T2-hyperintense WM LV** | **NBV** | **NGMV** | **NWMV** | **Thalamic volume** | **nMUCCA** |
| --- | --- | --- | --- | --- | --- | --- | --- |
| **MS patients with low aerobic capacity (31)** | **EDSS**  **r (p-value)** | 0.116 (0.534) | 0.146 (0.434) | 0.034 (0.856) | 0.138 (0.459) | -0.121 (0.517) | **-0.536 (0.002)** |
|  | **T25FWT**  **r (p-value)** | 0.128 (0.246) | 0.215 (0.246) | 0.066 (0.725) | 0.162 (0.384) | -0.078 (0.675) | -0.338 (0.063) |
|  | **9-HPT**  **r (p-value)** | 0.341 (0.060) | -0.253 (0.169) | **-0.378 (0.036)** | -0.133 (0.475) | **-0.525 (0.002)** | -0.252 (0.171) |
| **MS patients with high aerobic capacity (20)** | **EDSS**  **r (p-value)** | 0.094 (0.694) | -0.382 (0.097) | -0.305 (0.190) | -0.299 (0.201) | -0.323 (0.165) | -0.008 (0.972) |
|  | **T25FWT**  **r (p-value)** | 0.284 (0.226) | -0.056 (0.816) | -0.137 (0.565) | -0.094 (0.693) | -0.153 (0.520) | 0.173 (0.466) |
|  | **9-HPT**  **r (p-value)** | 0.161 (0.498) | -0.414 (0.070) | -0.215 (0.363) | -0.403 (0.078) | -0.316 (0.175) | -0.077 (0.748) |

Abbreviations: 9-HPT=nine-hole peg test; EDSS=expanded disability status scale; MS=multiples sclerosis; n=number; NBV=normalized brain volume; NGMV=normalized grey matter volume; nMUCCA= normalized mean upper cervical cord area; NWMV= normalized white matter volume; r=r-value; T25FWT= timed 25-foot walk test; WM LV=T2-hyperintense white matter lesion volume.
